# Genetic Analysis of *RAB39B* in an Early-Onset Parkinson's Disease Cohort

**DOI:** 10.3389/fneur.2020.00523

**Published:** 2020-06-26

**Authors:** Yujing Gao, Gabrielle R. Wilson, Nicholas Salce, Alexandra Romano, George D. Mellick, Sarah E. M. Stephenson, Paul J. Lockhart

**Affiliations:** ^1^Bruce Lefroy Centre for Genetic Health Research, Murdoch Children's Research Institute, Melbourne, VIC, Australia; ^2^Department of Paediatrics, University of Melbourne, Melbourne, VIC, Australia; ^3^Victorian Clinical Genetics Services, Murdoch Children's Research Institute, Melbourne, VIC, Australia; ^4^Griffith Institute for Drug Discovery (GRIDD), Griffith University, Nathan, QLD, Australia

**Keywords:** parkinson's disease, RAB39B, DNA polymorphisms, gene dosage, copy number variation

## Abstract

Pathogenic variants in the gene encoding RAB39B, resulting in the loss of protein function, lead to the development of X-linked early-onset parkinsonism. The gene is located within a chromosomal region that is susceptible to genomic rearrangement, and while an increased dosage of *RAB39B* was previously associated with cognitive impairment, the potential role of dosage alterations in Parkinson's disease (PD) remains to be determined. This study aimed to investigate the contribution of the genetic variation in RAB39B to the development of early-onset PD. We performed gene dosage studies and sequence analysis in a cohort of 176 individuals with early-onset PD (age of onset ≤ 50 years) of unknown genetic etiology. An assessment of the copy number variation over both coding exons and the 3′ untranslated region (UTR) of *RAB39B* did not identify any alterations in gene dosage. An analysis of the UTRs identified two male individuals carrying single, likely benign, nucleotide variants in the 3′UTR (chrX:154489749-A-G and chrX:154489197-T-G). Furthermore, one novel variant of uncertain significance was identified in the 5′UTR, 229 bp upstream of the start codon (chrX:154493802-C-T). *In silico* analyses predicted that this variant disrupts a highly conserved transcription factor binding site and could impact *RAB39B* expression. The results of this study do not support a significant role for genetic variation in *RAB39B* as contributing to early-onset PD but do highlight that additional molecular studies are required to determine the mechanisms regulating *RAB39B* expression and their association with the disease. Genetic investigations in larger parkinsonism/PD cohorts and longitudinal studies of individuals with cognitive impairment due to an altered dosage of *RAB39B* will be required to fully delineate the contribution of RAB39B to parkinsonism.

## Introduction

Parkinson's disease (PD) is a common neurodegenerative condition that manifests with a spectrum of motor symptoms including tremor, rigidity, bradykinesia, and gait disturbances. PD can be classified according to initial clinical presentation as early-onset PD (<50 years) or late-onset PD (>60 years). Despite a difference in disease onset, a post-mortem examination of the central nervous system in both classifications demonstrates the hallmark pathological features of the disease, including neuron loss in the substantia nigra *pars compacta* and the presence of intraneuronal α-synuclein (α-syn)-positive inclusions, termed as Lewy bodies.

Currently, the molecular mechanisms underlying the development and the progression of PD remain largely unknown, and most disease cases are idiopathic. However, in a subset of ~10% of cases, the disease etiology is genetic—the result of a monogenic mutation (1). Pathogenic variants in PD-associated genes can be point mutations or small in/dels that affect protein function or gene expression or can be larger copy number variants (CNV) that impact gene dosage. For example, protein-disrupting mutations and gene dosage alterations, which do not encompass the entire gene and result in loss of function, are an important mutation mechanism in recessive *parkin*-mediated PD (2). Similarly, multiplication of the entire gene encoding α-syn (SNCA), with associated increased dosage, expression, and elevated SNCA steady-state level, correlates with severity and disease progression in dominant PD (3–5). Genome-wide association studies have also identified additional risk loci contributing to the burden of the disease, including susceptibility alleles that can modulate the risk of developing PD through dysregulated gene expression. For example, the non-coding polymorphisms of the *SNCA* locus that impact promoter or enhancer activity correlate with a strong risk of developing sporadic PD (6–8).

Loss-of-function mutations in *RAB39B* were originally identified in two independent families who displayed the clinical features of early-onset Parkinson's disease (EOPD) with non-progressive intellectual disability and macrocephaly (9). RAB39B is a member of the RAB GTPase family with a putative role in vesicle trafficking. Several subsequent studies of the coding sequence and the splice junctions of *RAB39B* in large PD cohorts failed to identify additional pathogenic mutations, suggesting that the single-nucleotide variants in *RAB39B* that directly disrupt protein function are a rare cause of PD (10–14). However, genetic validation of the gene has been established by the identification of six additional causal *RAB39B* mutations, to date, in unrelated PD patients and families [reviewed in Ciammola et al. (15)]. Notably, a pedigree of European origin carrying a missense mutation in *RAB39B* (c.574G>A, p.G192R) manifested X-linked dominant PD in males, but the heterozygous females presented with later-onset parkinsonism and no intellectual disability (16). This potentially reduced penetrance in females suggests that the relative level of *RAB39B* expression may have an impact on the clinical presentation of PD.

*RAB39B* is located at Xq28 in a region flanked by low-copy repeats, making it susceptible to chromosomal aberrations mediated by a non-allelic homologous recombination. Indeed duplications at the Xq28 region, including the genes methyl CpG-binding protein 2 and GDP dissociation inhibitor 1, are frequently observed in males with intellectual disability and brain malformations (17). A single study investigating *RAB39B* copy number in a familial Chinese PD cohort (*n* = 195) did not identify any cases with dosage alterations (12). However, duplication and triplication of *RAB39B* have been previously reported to be associated with the development of X-linked intellectual disability (XLID) in male children (18, 19). It was not reported if the affected individuals presented with a movement disorder at the time of assessment.

The collective results, to date, have implicated *RAB39B* in the development of EOPD and parkinsonism. Although an altered dosage of *RAB39B* has been reported to cause XLID, it has not been associated with the development of PD to date. To further investigate the potential role of RAB39B in PD, we screened an EOPD cohort for CNV that could lead to an altered dosage of the gene. In addition, we performed sequence analysis of the untranslated regions (UTR) and immediately upstream of the putative transcription start site (TSS) to identify variants with the potential to dysregulate *RAB39B* expression.

## Materials and Methods

### Patient Samples

Prior to commencing the study, appropriate institutional ethics approval and informed consent from patients were obtained. Genomic DNA isolated from the whole blood of 232 individuals diagnosed with EOPD (onset ≤ 50 years) was made available by author GDM. This EOPD cohort, consisting of 71 females and 161 males with mean age of onset of 42.7 ± 6.5 years, comprises participants in the Queensland Parkinson's Project in Queensland, Australia (20) and is representative of a Caucasian population. All patient DNA samples were collected under protocols approved by the Griffith University Human Research Ethics Committee (Project ESK/04/11/HREC). The samples were previously sequenced to exclude mutations in known PD-associated genes, including *SNCA* (MIM 163890), *PARK2* (MIM 602544), *DJ1* (MIM602533), *PINK1* (MIM 608309), and *LRRK2* (MIM 609007). The samples were also previously screened for variants in the coding region of *RAB39B* (MIM 300774). A subset of the cohort (176 individuals, consisting of 58 females and 118 males with mean age of onset of 42.6 ± 6.5 years) was utilized in this study.

### Sequencing

**Figure 1:**
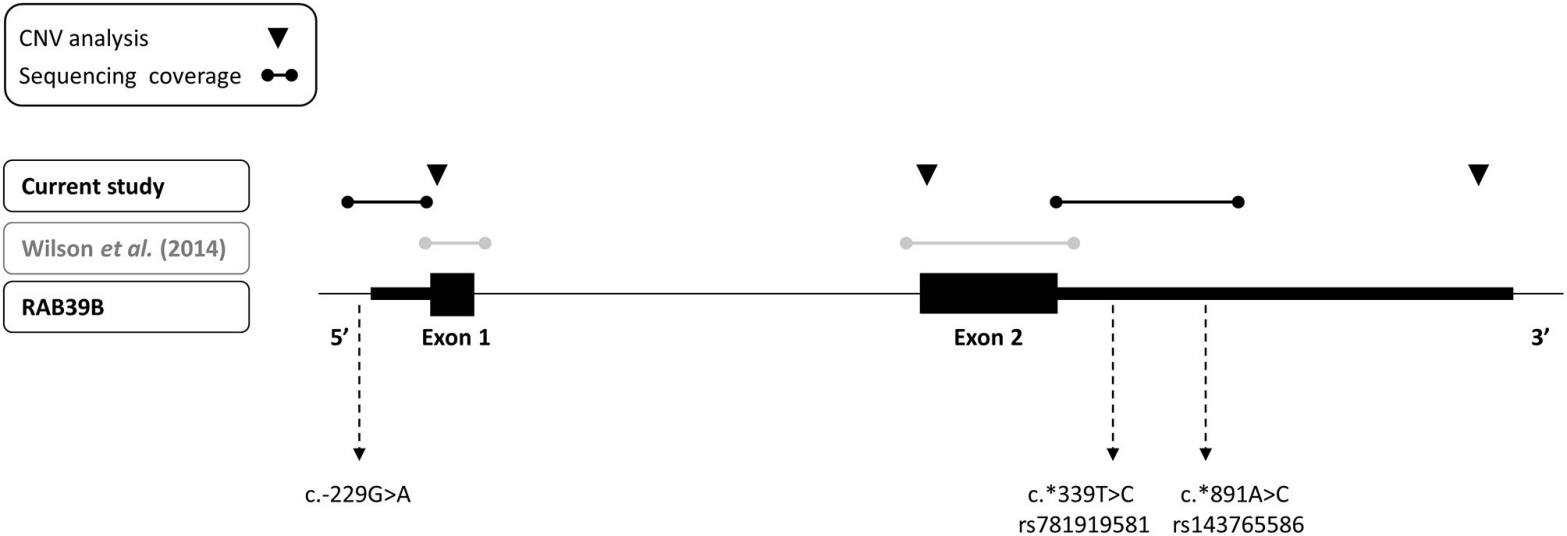
Depiction of sequencing and copy number variant analyses performed for *RAB39B* in this study (black text) and previously (gray text) of an early-onset Parkinson's disease cohort. The three variants identified in the study are indicated.

We amplified genomic DNA corresponding to regions of the upstream regulatory region, the 5′UTR and the 3′UTR of *RAB39B*, using the primers detailed in Table 1 and Figure 1. Sanger sequencing was performed using Big Dye Terminator v3.1 (Applied Biosystems, 4336697), according to the manufacturer's instructions, on 3730 Genetic Analyzer platform (Applied Biosystems). The sequences were aligned and analyzed using Sequencher 5.0 software (Genecodes). The detected variants were annotated using Varsome (https://varsome.com/) and filtered with GnomAD (https://gnomad.broadinstitute.org/). The variants not present in GnomAD were considered as novel. The pathogenicity of the variants was predicted using Combined Annotation Dependent Depletion (CADD) (https://cadd.gs.washington.edu/snv) and Deleterious Annotation of genetic variants using Neural Networks (DANN) (21), two *in silico* prediction tools designed to annotate both coding and no-coding variants. The reference cDNA and genomic sequences utilized for *RAB39B* were NM_171998.4 and GRCh37/hg19, respectively. The novel variants identified in this study have been submitted to the LOVD gene-specific database for *RAB39B* (https://www.lovd.nl/).

**Table 1:** Sequencing primers.

**Primer name**	**Sequence**	**Amplicon size**
hRAB39B 5′UTR F	TGGCAGTTTGAACGACAGAG	397 bp
hRAB39B 5′UTR R	GCTCTGCAGGTCTCCTTGG	
hRAB39B 3′UTR 1F	CATGCTCTCCTACTTGAACTGAA	1,000 bp
hRAB39B 3′UTR 1R	CCTGGCCAAGTGATTTTCAT	

### CNV Analysis

We performed an analysis of *RAB39B* CNV by quantitative real-time PCR (RT-PCR), utilizing commercially available Taqman assays interrogating exon 1, exon 2, or the 3′UTR of *RAB39B* (Life Technologies, Hs00817269_cn, Hs00745075_cn, and Hs02637133_cn, respectively; Figure 1). The reactions were duplexed with the human RNaseP copy number reference assay (Life Technologies, 4403326) and 10–20 ng gDNA amplified on a LightCycler LC480 II (Roche) according to the manufacturer's instructions. Each sample was assessed in triplicate. The threshold cycle was determined using LightCycler LC480 software 1.5.1.62 SP2, and *RAB39B* copy number was calculated using the ΔΔCT method.

## Results

**Figure 2:**
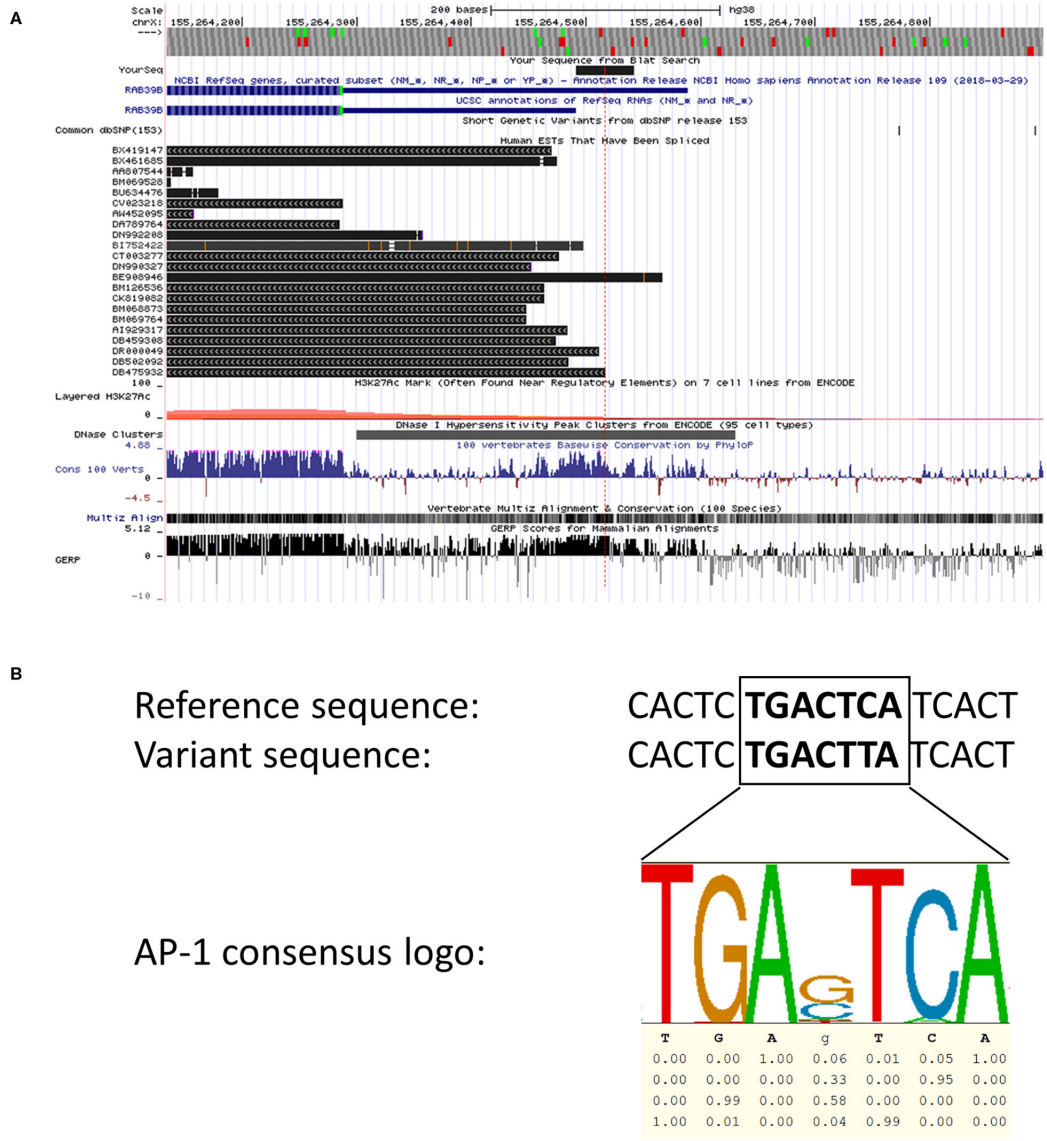
*In silico* analyses of the 5′ region of *RAB39B*. **(A)** Screenshot of the UCSC browser (hg38, chrX:155,264,156-155,264,850) examining the 5′ region of *RAB39B*. The location of the chrX:154493802-C-T variant is depicted by the red dotted line. The blue sequences represent Refseq NM_171998.3 (outdated) and NM_171998.4, respectively. Spliced human expressed sequence tags are shown in black. The lower tracks demonstrate that the variant is located within a region of DNase 1 sensitivity and displays high vertebrate conservation compared to immediate flanking sequence. The final track demonstrating GERP scores represents an analysis of the corresponding sequence using the hg19 dataset. **(B)** An alignment of the 5′ region of *RAB39B* showing the reference genomic sequence (top) with the variant sequence (middle). The predicted AP-1 transcription factor binding site is in bold highlight. The sequence logo (bottom) generated from ENCODE data demonstrates the core AP-1 consensus sequence and the conservation of each nucleotide.

We screened for CNVs over both coding exons of RAB39B and the 3′UTR by quantitative RT-PCR (Figure 1) but did not identify any variations in *RAB39B* exon or gene dosage. In addition, we analyzed 404 bp of sequence upstream of the initiating codon and 1,021 bp downstream of the termination codon for sequence variants in *RAB39B* in 176 individuals with EOPD of unknown genetic etiology (Figure 1). We identified three male individuals carrying single-nucleotide variants. One variant of uncertain significance (chrX:154493802-C-T) was identified in the 5′UTR, 229 bp upstream of the ATG start codon and close to the predicted TSS of Refseq NM_171998.4 (Figure 2A), of a male patient with a disease onset age of 50 years. This nucleotide is highly conserved (GERP 4.6) and the variant is predicted to disrupt a consensus activator protein-1 (AP-1) transcription factor binding site located within a DNase 1 hypersensitive peak (Figure 2B). This is a novel variant not previously identified in GnomAD, with *in silico* support of pathogenicity utilizing DANN (score 0.98) and CADD (score 21.2). Due to the study design of the Queensland Parkinson's Project (22), we were unable to test if the variant was *de novo* or perform functional studies of the variant in patient-derived cells. No intellectual issues were reported at the time of patient examination and there was no familial history of parkinsonism. Two likely benign variants were identified in the 3′UTR region of *RAB39B*. One variant was identified in a male patient with disease onset age of 48 years (NM_171998.4:c.^*^339T>C; chrX:154489749-A-G). This rare variant (rs781919581) has an average allele frequency of 0.00086 in GnomAD, with DANN and CADD scores of 0.75 and 5.29, respectively. The second variant was identified in a male patient with a disease onset age of 49 years (NM_171998.4:c.^*^891A>C; chrX-154489197-T-G). This rare variant (rs143765586) has an average allele frequency of 0.00087 in GnomAD, with DANN and CADD scores of 0.66 and 1.67, respectively.

## Discussion

*RAB39B* is a member of the RAB GTPase family with a putative role in vesicle trafficking in neurons. While there is considerable genetic and functional evidence demonstrating the loss of function mutations that cause an early-onset familial parkinsonian disorder in males, a potential broader role in idiopathic PD remains to be fully tested. Previously, we investigated a Caucasian EOPD cohort (*n* = 187) for alterations in the coding regions of *RAB39B* and found no variants of significance (9). In this study, we investigated a subset of this EOPD cohort for CNVs and non-coding variants that could potentially result in the dysregulated expression of *RAB39B*. Although the non-coding variants may not directly impact protein function, they can alter the protein levels in neurodegenerative diseases such as PD by modulating mRNA synthesis, stability, localization, and translation. Non-coding polymorphisms in *SNCA, PARKIN*, and *DJ1* have all been previously identified to be associated with the development of PD in cohort screens (23–27). For example, while protein-disrupting mutations in *parkin* are a common cause of recessive EOPD (28), the variants in the promoter/5′UTR region that affect *parkin* expression are associated with idiopathic PD (29).

We did not identify any CNV alterations in the 176 samples analyzed, suggesting that increased *RAB39B* dosage may not be associated with EOPD. While screening additional large cohorts will further test this hypothesis, longitudinal studies of individuals with XLID secondary to duplication and triplication of *RAB39B* (18, 19) will also inform whether an increased dosage of RAB39B can cause a parkinsonian phenotype. Given that the PD phenotype associated with the loss of RAB39B function appears to manifest later in life compared to intellectual disability [>20 years; (15)], it is probable that if the affected individuals are going to develop parkinsonism, it will be at a later age than the time of report.

Screening of the non-coding regions of *RAB39B* revealed three variants, one immediately proximal to the TSS and two within the 3′UTR. Both UTR variants were classified as likely benign according to the ACMG guidelines (30). In contrast, an *in silico* analysis identified that the upstream variant chrX:154493802-C-T was novel, with predictions supportive of pathogenicity. Our analysis of both expressed sequence tags and genomic conservation around the variant suggests that it disrupts a highly conserved AP-1 transcription factor binding site. We hypothesize that this motif is important for regulating the *RAB39B* expression, and the variant likely downregulates the expression by preventing the binding of important transcription factors such as AP-1. The AP-1 family of transcriptional factors can modulate a wide range of molecular functions, one of which is neuronal plasticity (31). The AP-1 regulation of neuron-enriched RAB GTPases has not been previously reported, although one study demonstrated that AP-1 can regulate *RAB11A* promoter activity and thus endosomal recycling (32). Interestingly, a phylogenetic analysis of the RAB GTPase family shows that RAB39 shares the most recent common ancestor with RAB11 (33), suggesting that the transcriptional regulation of some RAB GTPases may be evolutionarily conserved. Currently, knowledge of the transcriptional regulation of *RAB39B* is lacking. Specifically, the promoter region, primary TSS, and important transcription factors for *RAB39B* have yet to be identified and functionally characterized. Therefore, while our analysis of the chrX:154493802-C-T variant is consistent with a potential effect on *RAB39B* expression, in the absence of functional validation, the significance of the variant remains uncertain.

Overall our results are consistent with previous reports suggesting that the genetic variation in *RAB39B* is a rare cause of EOPD. A genetic analysis of the UTRs and the regulatory regions of *RAB39B* has not been reported previously; our identification of a novel 5′ variant, with *in silico* predictions supporting pathogenicity, warrants further investigation. Moreover, a recent study in a small cohort of individuals with idiopathic PD suggested that steady-state levels of RAB39B in brain tissue might be decreased (34). Therefore, further genetic and functional studies are required to determine the consequences of dysregulated *RAB39B* expression and test its potential role as a susceptibility gene associated with PD or parkinsonism more broadly.

## Data Availability Statement

Details of variants identified in this study have been submitted to the LOVD gene specific database for RAB39B https://databases.lovd.nl/shared/genes/RAB39B.

## Ethics Statement

The studies involving human participants were reviewed and approved by Griffith University human research ethics committee (project ESK/04/11/HREC). The patients/participants provided their written informed consent to participate in this study.

## Author Contributions

YG and GW contributed to study design, execution, and data analysis. NS and AR contributed to study execution and data analysis. GM provided materials and contributed to study design. SS contributed to study design and data analysis. PL designed and funded the study and contributed to study conception and data analysis. YG and PL wrote the first draft of the manuscript. All the authors contributed to manuscript revision and read and approved the submitted version.

## Conflict of Interest

The authors declare that the research was conducted in the absence of any commercial or financial relationships that could be construed as a potential conflict of interest. The handling editor declared a past collaboration with the authors.
